# Physical activity guidelines and promotion: An online survey of United Kingdom’s prosthetic rehabilitation healthcare professionals

**DOI:** 10.1177/0309364620920109

**Published:** 2020-05-24

**Authors:** Sarah Deans, Alison Kirk, Anthony McGarry, David Rowe

**Affiliations:** 1 Deans Civil Engineering, Glasgow, UK; 2 National Centre for Prosthetics and Orthotics, Department of Biomedical Engineering, University of Strathclyde, Glasgow, UK; 3 Physical Activity for Health, School of Psychological Sciences and Health, University of Strathclyde, Glasgow, UK

**Keywords:** Prosthetics, rehabilitation of amputees, rehabilitation of prostheses users, rehabilitation, education

## Abstract

**Background:**

Healthcare professionals play a key role in supporting physical activity participation for people with lower limb absence.

**Objectives:**

The objectives of this study were to survey healthcare professionals’ views of people with lower limb absence in the United Kingdom, explore their awareness and knowledge of physical activity recommendations and investigate their current and desirable practice towards physical activity promotion.

**Study design:**

Cross-sectional study.

**Methods:**

Potential participants were identified from open-access health-related databases, educational institution databases, and the authors’ professional networks. An online 40-item questionnaire was distributed electronically and by post. Survey items were multiple choice, Likert-type scale or open-ended questions to explore the characteristics of healthcare professionals, awareness/knowledge of physical activity guidelines, current and desired practice and views on physical activity promotion.

**Results:**

In total, 106 people responded. Physiotherapists had greater awareness/knowledge of physical activity guidelines compared to prosthetists/orthotists and other respondents. Awareness/knowledge of guidelines decreased as age, experience and time since qualification increased. The most common source of knowledge was self-directed learning.

**Conclusion:**

Continuing and improving education on the content of physical activity guidelines may be helpful for healthcare professionals in promoting physical activity to those with lower limb absence.

**Clinical relevance:**

This research aims to inform prosthetic rehabilitation professionals and academics about an under-researched area within physical activity for health. This knowledge could help develop interventions with the aim of improving physical activity promotion and participation, and ultimately the health and well-being of people with limb absence.

## Background

In the United Kingdom, during 2011–2012, 5906 people were referred to regional prosthetic rehabilitation services with upper and lower limb absence.^1^ The majority had lower limb absence (91%), were male (70%), were over 54 years of age (70%) and were referred due to compromised vascular causes (68%).^1^ These statistics are comparable to data from the United States; however, may not be representative of lower income countries.^2^ Risk factors for peripheral arterial disease and subsequent lower limb amputation include the following: age, gender, cardiovascular disease, smoking, hypertension, obesity and lack of physical activity.^2^ Lack of physical activity is a potentially modifiable factor that could be addressed by the rehabilitation team.

Socialised prosthetic rehabilitation is generally delivered in the United Kingdom by a consultant-led service supported by Health and Care Professions Council (HCPC)-registered prosthetists, physiotherapists, occupational therapists, and clinical nurse specialists.^3^ Regional National Health Service (NHS) Disablement Services Centres exist throughout England, Northern Ireland, Scotland and Wales with the number of prosthetists working at each centre varying from 1 to 10 depending on the regional patient case-load. Each Disablement Service Centre will have access to physiotherapists who will be specialists in amputation physiotherapy rehabilitation. Although it is possible to retrieve data on the number and gender of prosthetists, orthotists and physiotherapists registered with HCPC, it is not possible to derive the proportion of these professional groups who specialise in prosthetic rehabilitation.^4,5^ In addition to socialised prosthetic care, there are a number of independent, privately owned rehabilitation facilities operating in the United Kingdom.^6^

People who engage in physical activity have a lower risk of developing diseases including cardiovascular diseases, type 2 diabetes and some cancers.^7^ Individuals who are physically active enjoy a range of benefits spanning physiological, emotional, cognitive and social categories. In addition, there are psychological and social benefits to be gained by people with limb absence who participate in physical activity.^8,9^ Current guidelines, published by the Chief Medical Officer of the United Kingdom, advise that adults should participate in at least 150 min of moderate-intensity physical activity a week, or an equivalent amount of vigorous-intensity physical activity.^10^ Muscle-strengthening activities should be included on at least 2 days, and the time spent being sedentary (sitting) should be minimised.^10^ At the time of writing, there are no known published physical activity guidelines for patients with limb absence. However, it is acknowledged that an evidence review has been published by Public Health England (PHE) that highlights a critical need for disabled adults to do more physical activity to improve their health.^11^ Furthermore, the Chief Medical Officer in the United Kingdom has made recommendations for physical activity to be more accessible and to support disabled people to become more active.^12^

Participation in physical activity declines with age and can also be affected by socioeconomic factors. Only around 20% of men and women aged between 65 and 74 years achieve the recommended physical activity levels, and this declines further to around 5%–10% of adults over the age of 75 years.^13,14^ Numerous barriers to promoting physical activity have been reported by exercise professionals, including lack of awareness, lack of knowledge of the content, and lack of time to do so within the patient consultation.^15,16^ People with physical disability experience difficulty with physical activity participation due to factors such as the management of the primary disabling condition and secondary complications, for example, diabetes contributing to limb absence.^17^ Programme factors such as compliance in a weekly exercise class may also be challenging. Personal factors such as being conscious of body image, and environmental factors such as inclement weather conditions may also impact negatively in participation.^17^ Patients can also perceive physical activity recommendations as not applicable to them or unrealistic in attainment. Instead patients place a high level of importance on maintaining strong relationships with family, friends and their peers.^9^

The authors propose that encouraging people with lower limb absence to maintain or increase physical activity levels and reduce sedentary behaviour is a critical component of care. It has been shown that healthcare professionals can have a key role in supporting this aspect of rehabilitation and ongoing treatment.^18,19^ However, limited research has been conducted to explore healthcare professionals’ awareness and knowledge of current physical activity recommendations, in addition to their current and desirable practice towards physical activity promotion. Indeed, the majority of people with lower limb absence are elderly with many comorbidities.^1^ It may be that other recommendations and guidelines may be more appropriate for this group. Furthermore, promoting physical activity within this population requires sound knowledge of comorbidities affecting health and how physical activity recommendations may be devised. Based on this, the study aims were as follows:

1. Explore prosthetic healthcare professionals’ awareness and knowledge of physical activity guidelines;
2. Investigate current and desired practice of promoting physical activity to people with limb absence;
3. Explore UK healthcare professionals’ views on physical activity promotion for people with limb absence.

## Methods

### Participants

Participants were rehabilitation medicine consultants, prosthetists, orthotists, physiotherapists, occupational therapists, nurses and clinical psychologists. Potential participants were identified from the following sources: open-access health-related databases, educational institution databases, and the authors’ professional networks. The number of people estimated to be working in the UK prosthetic rehabilitation environment at the time of recruitment was thought to be in the region of 370.^20^ It was hoped that the majority of these would have been made aware of the survey through this recruitment drive. Individuals were contacted in the four UK home countries by email or post. These were as follows: UK Disablement Service Centre Managers (*n* = 53), private prosthetic service providers (*n* =. 6), professional associations of healthcare professionals (*n* *=* 4) and support organisations (*n* *=* 4). All participants gave written informed consent.

### Measurement instrument

A 40-item online survey was developed by the authors and distributed using Qualtrics^®^, Version 2016 (Qualtrics, Provo, UT). Supplemental File 1 contains the survey as it appeared online to participants. As recommended by Stone in 1993,^21^ pilot testing of the survey was conducted with 20 volunteers who collectively had expertise in prosthetics and orthotics rehabilitation, physical activity behaviour, physical activity measurement and survey design. The volunteers were recruited from one of the author’s professional networks. Feedback from pilot testing prompted adjustments to be made to improve survey continuity, logic and readability. The content of certain questions pertaining to exercise intensity was also reworded. The time taken to complete the questionnaire was noted (from the online software output) to be on average around 10 min.

Survey questions were divided into four sections:

Part I had 15 questions measuring healthcare professionals’ awareness and existing knowledge of current weekly UK physical activity guidelines;
Part II contained 11 questions assessing healthcare professionals’ views on their current practice of physical activity promotion;
Part III contained seven questions measuring healthcare professionals’ attitudes and beliefs on desirable practices for physical activity promotion;
Part IV contained six demographic questions and one final open-ended question to offer respondents an opportunity to comment on relevant topics not covered elsewhere in the survey.

Questions in Parts I, II and III were multiple choice style or scored on a 5-point Likert-type scale. A knowledge score was constructed from the responses to questions about the guidelines (Supplemental File 2). Each correct answer increased the respondent’s score by a single point. Physical activity descriptors used in the survey were taken from the report on physical activity from the Chief Medical Officers of the four home countries of the United Kingdom.^10^ The survey was available for 6 months.

### Data screening and analysis

Qualtrics data were exported to SPSS Version 22 (IBM Corp., Armonk, NY). Descriptive statistics were calculated and professional titles were aggregated into three groups (prosthetists/orthotists, physiotherapist, and other) to allow sufficient sample for comparison analysis. Data analysis consisted of the following: chi-square for comparing frequencies of nominal level data; one-way analysis of variance for comparing interval/ratio level data across the three groups, with least significance difference follow-up tests for between-group comparison; and Pearson *r* for relationships between continuous demographic data and primary outcome variables. The critical alpha used to determine significance was .05.

## Results

### Participant characteristics

A total of 106 respondents completed the survey. Participant characteristics are presented in Table 1.

**Table 1.** Characteristics of survey participants.

	All respondents		Prosthetist/orthotist		Physiotherapist		Other	
	*N*	(%)	*n*	(%)	*n*	(%)	*n*	(%)
Professional affiliation								
Prosthetist/orthotist	34	(32.4)	34	(63.0)				
Prosthetist	19	(18.1)	19	(35.2)				
Orthotist	1	(1.0)	1	(1.9)				
Physiotherapist	33	(31.4)			33	(100.0)		
Medic	3	(2.9)					3	(16.7)
Nurse	4	(3.8)					4	(22.2)
Other	11	(10.5)					11	(61.1)
Missing	1^a^							
Gender								
Male	35	(33.0)	25	(46.3)	3	(9.1)	6	(33.3)
Female	71	(67.0)	29	(53.7)	30	(90.9)	12	(66.7)
Age (years)								
20–30	19	(17.9)	11	(20.4)	5	(15.2)	2	(11.1)
31–40	34	(32.1)	17	(31.5)	13	(39.4)	4	(22.2)
41–50	29	(27.4)	14	(25.9)	11	(33.3)	4	(22.2)
⩾51	24	(22.6)	12	(22.2)	4	(12.1)	8	(44.4)
Years qualified								
0–10	28	(26.7)	15	(28.3)	9	(27.3)	3	(16.7)
11–20	42	(40.0)	20	(37.7)	13	(39.4)	9	(50.0)
21–30	23	(21.9)	11	(20.8)	10	(30.3)	2	(11.1)
31–40	12	(11.4)	7	(13.2)	1	(3.0)	4	(22.2)
Missing	1		1^b^					
Years working in clinical practice								
0–10	31	(29.5)	18	(33.3)	9	(27.3)	3	(17.7)
11–20	40	(38.1)	20	(37.0)	12	(36.4)	8	(47.1)
21–30	22	(21.0)	9	(16.7)	11	(33.3)	2	(11.8)
31–40	12	(11.4)	7	(13.0)	1	(3.0)	4	(23.5)
Missing	1						1^c^	
Geographical location of usual place of work								
England	49	(48.0)	24	(47.1)	19	(59.4)	5	(27.8)
Northern Ireland	2	(2.0)	1	(2.0)	1	(3.1)		
Scotland	47	(46.1)	24	(47.1)	11	(34.4)	12	(66.7)
Wales	4	(3.9)	2	(3.9)	1	(3.1)	1	(5.6)
Missing	4		3^d^		1^e^			
Total	106	(100)	54	(100)	33	(100)	18	(100)

a One other respondent did not declare their professional title.

b One prosthetist/orthotist did not declare the number of years qualified.

c One other respondent did not declare the number of years qualified.

d Three prosthetist/orthotists did not declare their geographical location.

e One physiotherapist did not declare their geographical location.

A total of 10.5% of respondents (*n* = 11) declared their professional title was something other than the six options available. These were coach (*n* = 1), occupational therapist (*n* = 3), podiatrist (*n* = 3), senior lecturer (*n* = 1), student orthotist (*n* = 1) and surgeon (*n* = 2). Similarities between professionals lay in age but not in gender. This is representative of the UK healthcare professional population working in rehabilitation medicine. There is a gender bias to more females working in the field of physiotherapy in the United Kingdom, and fewer female prosthetists than male working in prosthetics.^4^

### Knowledge and understanding of physical activity guidelines

Overall, 60.4% of all respondents were aware of the guidelines. Table 2 details the awareness of the presence and content of the guidelines, and the source of awareness of the content.

**Table 2.** Awareness of existence and awareness of contents of physical activity guidelines and source of awareness.

Question	Statistic	Prosthetist/orthotist		Physiotherapist		Other		NA	*df*	*t*	*p*	Fisher’s exact probability
		Yes	No	Yes	No	Yes	No					
Are you aware there are physical activity guidelines?	*N* (%)	28 (51.9)	26 (48.2)	26 (78.8)	7 (21.2)	10 (55.6)	8 (44.4)		2	6.51	.04^a^	.04
Are you aware of the content of physical activity guidelines?	*N* (%)	17 (60.7)	11 (39.3)	19 (73.1)	7 (26.9)	8 (80.0)	2 (20.0)	41	2	1.66	.44^b^	.51
Source of awareness of physical activity guidelines												
On-line learning	*n* (%)	6 (21.4)	22 (78.6)	7 (26.9)	19 (73.1)	3 (30.0)	7 (70.0)	41	2	0.38	.83	.80
Higher education course	*n* (%)	4 (14.3)	24 (85.7)	3 (11.5)	23 (88.5)	0 (0.0)	10 (100.0)	41	2	1.56	.46	.67
Work-based seminar	*n* (%)	5 (17.9)	23 (82.1)	1 (3.9)	25 (96.2)	0 (0.0)	10 (100.0)	41	2	4.34	.11	.15
Self-directed learning	*n* (%)	7 (25.0)	21 (75.0)	11 (42.3)	15 (57.7)	1 (10.0)	9 (90.0)	41	2	4.14	.13	.16
Published articles	*n* (%)	6 (21.4)	22 (78.6)	7 (26.9)	19 (73.08)	4 (40.0)	6 (60.0)	41	2	1.31	.52	.52
Other source	*n* (%)	5 (17.9)	23 (82.1)	2 (7.7)	24 (92.3)	2 (20.0)	8 (80.0)	41		1.50	.47	.50

*df*: degree of freedom.

a Significant difference between groups in awareness of guideline existence.

b No significant difference between professional groups in awareness of guideline content.

The knowledge score yielded the following results from the responses to questions about the guidelines (Supplemental File 2). Respondents scored 6.42 out of 11, with significant variation (*F*(2, 102) = 4.32, *p* = .016) across professional groups. Physiotherapists scored 7.00 out of 11, prosthetists/orthotists scored 6.24, and other 5.89.

There was a likelihood that participants who had been aware of the content of the guidelines had higher knowledge scores. This was found for all groups (*t*(104) = 5.24, *p* < .000), in prosthetist/orthotists (*t*(52) = 3.89, *p* = .0003) and in physiotherapists (*t*(31 = 2.73, *p* = .011). However, no significant difference was detected in the other professionals (*t*(16) = .80, *p* = .43). Weak relationships existed between knowledge and age (*r* = –.23, *p* = .019). A similar trend existed for knowledge and years qualified (*r* = –.16, *p* = .10), and knowledge and years working in clinical practice (*r* = –.19, *p* = .06). As age, years qualified and years working in clinical practice increased, knowledge of physical activity guidelines decreased. A similar, but non-significant trend was found for each profession group with negative correlations between knowledge, age, year qualified and years working in clinical practice.

### Views on current physical activity promotion

The survey examined respondents’ current practice in promoting physical activity in the prosthetic rehabilitation setting. In summary, physiotherapists

- Promoted physical activity to patients more than both prosthetist/orthotists and other professionals (*F*(2, 101) = 10.32, *p* < .001) and were more likely to state that they had adequate knowledge to promote physical activity to patients than prosthetist/orthotists (*F*(2, 101) = 8.65, *p* < .001);
- Were more likely to state that they were confident in promoting physical activity to patients than prosthetist/orthotists (*F*(2, 101) = 8.25, *p* < .001);
- Were more likely to have undertaken pre-qualification (*F*(2, 100) = 16.95, *p* < .001) and post-qualification (*F*(2, 99) = 6.65, *p* = .002) learning on the topic of physical activity promotion;
- Were shown to be significantly more likely than either of the other two professional groupings to say that their professional association encouraged them to promote physical activity.

The significance is noteworthy in questions 17 and 18 ‘I enjoy promoting physical activity to patients’ (*F*(2, 101) *=* 2.74, *p* = .069) and ‘I have time to promote physical activity to patients’ (*F*(2, 100) *=* 2.67, *p* = .074). Importantly, one question in this group showed no significant differences between groups: ‘I discuss physical activity promotion with other health & social care professionals’ (*F*(2, 100) *=* 1.49, *p* = .230). Levels of discussion about physical activity promotion between professionals were found to be similar. Table 3 details the results of analysis on both respondents’ current and desirable views.

**Table 3.** Current and desired practice related to physical activity promotion.

Question	Statistic	All	Prosthetist/orthotist	Physiotherapist	Other	*df* (between, within groups)	*F*	*p*	Post hoc tests (LSD)		
									Prosthetist/orthotist compared to physiotherapist	Prosthetist/orthotist compared to other	Physiotherapist compared to other
Current practice											
Q16 I promote physical activity to patients	*M*	3.91	3.57	4.48	3.88	2, 101	10.32	<.000	*		*
	SD	0.99	0.98	0.62	1.11						
	*n*	104	54	33	17						
Q17 I enjoy promoting physical activity to patients	*M*	3.97	3.78	4.24	4.06	2, 101	2.74	.0693	*		
	SD	0.93	0.92	0.75	1.14						
	*n*	104	54	33	17						
Q18 I have time to promote physical activity to patients	*M*	3.57	3.52	3.82	3.25	2, 100	2.67	.0741			*
	SD	0.86	0.91	0.58	1.06						
	*n*	103	54	33	16						
Q19 I have adequate knowledge to be able to promote physical activity to patients	*M*	3.70	3.43	4.15	3.71	2, 101	8.65	.0003	*		
	SD	0.85	0.84	0.57	0.99						
	*n*	104	54	33	17						
Q20 I am confident about promoting physical activity to patients^a^	*M*	3.83	3.52	4.27	3.94	2, 101	8.25	.0005	*		
	SD	0.91	0.88	0.63	1.09						
	*n*	104	54	33	17						
Q21 Other health and social care professionals should promote physical activity to patients	*M*	4.21	4.19	4.24	4.22	2, 102	0.07	.9347			
	SD	0.72	0.70	0.75	0.73						
	*n*	105	54	33	18						
Q22 I discuss physical activity promotion with other health and social care professionals	*M*	**3.17**	**3.04**	**3.25**	**3.47**	2, 100	1.49	.220			
	SD	0.95	0.95	0.88	1.07						
	*n*	103	54	32	17						
Q23 My workplace management expects me to promote physical activity to patients	*M*	**3.37**	**2.85**	**4.06**	**3.65**	2, 101	11.66	.000	*	*	
	SD	1.28	1.19	1.03	1.32						
	*n*	104	54	33	17						
Q24 My professional association encourages me to promote physical activity to patients	*M*	**3.53**	**2.80**	**4.67**	**3.65**	2, 101	28.39	.000	*	*	*
	SD	1.39	1.28	0.60	1.37						
	*n*	104	54	33	17						
Q25 I have undertaken pre-qualification learning on the topic of physical activity promotion^a^	*M*	**1.93**	**1.41**	**2.91**	**1.76**	2, 100	16.95	.000	*		*
	SD	1.33	0.88	1.47	1.30						
	*n*	103	54	32	17						
Q26 I have undertaken or am undertaking post-qualification learning on the topic of physical activity promotion^a^	*M*	**2.01**	**1.58**	**2.63**	**2.18**	2, 99	6.65	.002	*		
	SD	1.36	1.05	1.45	1.63						
	*n*	102	53	32	17						
Desired practice											
Q27 I should promote physical activity to patients	*M*	**4.45**	**4.31**	**4.70**	**4.39**	2, 102	4.48	.0136	*		
	SD	0.60	0.58	0.53	0.70						
	*n*	105	54	33	18						
Q28 My workplace management should expect me to promote physical activity to patients	*M*	**4.22**	**4.07**	**4.59**	**4.00**	2, 101	5.71	.0045	*		*
	SD	0.79	0.70	0.67	1.03						
	*n*	104	54	32	18						
Q29 My professional association should encourage me to promote physical activity to patients	*M*	**4.28**	**4.02**	**4.73**	**4.22**	2, 102	11.83	<.0001	*		*
	SD	0.73	0.71	0.52	0.73						
	*n*	105	54	33	18						
Q30 Other health and social care professionals should promote physical activity to patients	*M*	**4.36**	**4.28**	**4.55**	**4.28**	2, 102	2.39	.0969	*		
	SD	0.59	0.53	0.62	0.67						
	*n*	105	54	33	18						
Q31 Pre-qualification health and social care students should be educated at higher education level on patient physical activity promotion	*M*	**4.18**	**4.09**	**4.42**	**4.00**	2, 101	2.45	0.0914			
	SD	0.79	0.77	0.75	0.84						
	*n*	104	53	33	18						
Q32 CPD courses should exist on patient physical activity promotion	*M*	**4.30**	**4.13**	**4.58**	**4.28**	2, 102	5.82	0.0040	*		
	SD	0.62	0.62	0.50	0.67						
	*n*	105	54	33	18						
Q33 I would attend patient physical activity promotion CPD courses if they were available	*M*SD*n*	**4.01**0.80105	**3.91**0.7154	**4.27**0.6333	**3.83**1.2018	2, 102	2.73	0.0698	*		

LSD: least significant difference; SD: standard deviation; *df*: degree of freedom; CPD: continuing professional development. Significance for bold values is p = 0.05

a Significant Levene test indicating variances (SDs) are not equal.

* indicates the overall significance between groups with significance p = 0.05

### Views on desirable physical activity promotion

The survey examined attitudes and beliefs regarding desirable practice among healthcare professionals when promoting physical activity to people with limb absence. Physiotherapists indicated more than the other two professional groups described that

- Physiotherapists should promote physical activity to patients (*F*(2, 102) = 4.48, *p* = .014);
- Workplace management should expect physiotherapists to promote physical activity to patients (*F*(2, 101) = 5.71, *p* = .005);
- Their professional association should encourage physiotherapists to promote physical activity to patients (*F*(2, 102) = 11.83, *p* < .001);
- Continuing professional development (CPD) courses should exist on patient physical activity promotion (*F*(2, 102) = 5.82, *p* = .004).

Finally, 29 respondents provided further personal views through text entry statements in an open-ended, unstructured question (Supplemental File 3). Four key themes emerged from these open-ended questions as follows: healthcare professionals being role models for an active lifestyle; the presence of comorbidities being a key barrier to physical activity participation; inadequate prosthetic provision being a barrier to participation; and an acknowledgement that healthcare professionals have a key role to play in physical activity promotion.

## Discussion

The aims of this study were to explore prosthetic healthcare professionals’ awareness and knowledge of physical activity guidelines, investigate current and desired practice of promoting physical activity to people with limb absence and explore UK healthcare professionals’ views on physical activity promotion for people with limb absence.

The research explores topics similar to other notable studies on attitudes, beliefs and current and desired practice in physical activity promotion^18,19,22–24^; however, it is unique in being focused towards physical activity promotion by healthcare professionals involved in the care of people with limb absence.

There was variation across professions for the degree of knowledge of the physical activity guidelines. Specifically, physiotherapists were most likely to be aware of the presence of the guidelines and better informed about the content than prosthetists/orthotists and other professionals. This may be attributed to the fact that within a UK prosthetic rehabilitation team, the role of the physiotherapist is to include exercise therapy in immediate pre- and post-operative rehabilitation.

Healthcare professionals who were more aware of the existence and the content of the guidelines had a better knowledge score performance. Overall, physiotherapists achieved higher scores than prosthetists/orthotists and other healthcare professionals. The knowledge questions that respondents had most difficulty answering were those enquiring about the number of minutes for which moderate and vigorous activities should be performed over a week. Healthcare professionals in this study underestimated the number of minutes. Conversely, the same healthcare professionals overestimated the number of days that people should engage in muscle strengthening and flexibility activities. An implication of this could be that physical activity could be promoted inaccurately and patients over-exert themselves to the detriment of their health. Furthermore, confusion seems to remain around important more detailed elements of the guidelines, for example, in relation to strength and flexibility training. Updates and changes to existing published international or national guidelines may serve to reinforce a mixed message and possibly confusing approach. The majority of professionals who offered comment in this survey reported anxiety in communicating physical activity benefits to patients living with comorbidity. Therefore, a case may be made for creating dedicated guidelines specifically for people with limb absence which includes guidance on how to advise those with comorbidities. Pre-existing guidelines, such as those for people with diabetes or cardiovascular disease,^25–28^ could be helpful in creating physical activity guidelines for people with lower limb absence. It is indeed a positive step for PHE and the Chief Medical Officer in the United Kingdom to introduce initiatives to support disabled adults to improve their health with fact sheets and infographics.^11,12,29^

The source of the survey respondents’ awareness of the existence and content of the guidelines appears to be self-directed in nature. A more formalised approach to dissemination of recognised guidelines for post-qualification professionals should be considered. This could take the form of position statements from professional associations, or bespoke CPD courses delivered in the workplace, online, or from external educational sources. Furthermore, there may be a case for including the topic of physical activity promotion in recognised guidelines such as The Amputee and Prosthetic Rehabilitation Standards.^3^

As age, experience and time since qualification increased, in general, knowledge score decreased. Even though a professional may be highly experienced, their knowledge of the latest guidelines may be more limited than someone who was recently qualified. This finding adds support to the idea that refresher courses could be beneficial in familiarising experienced professionals with the latest clinical developments and practices.

A positive outcome of this study was the finding that prosthetic rehabilitation healthcare professionals are promoting physical activity to people with limb absence and feel they have the knowledge to do so. However, although most survey respondents (90.4%) felt they had the knowledge to advise patients on physical activity, respondents only achieved a mean knowledge score of 6.42 out of 11 knowledge items (i.e. 58.4% correct). Another study of general practitioners showed 77% of respondents felt they had sufficient knowledge to advise patients on physical activity, yet only 22.4% of respondents tried to encourage patients to increase their physical activity.^30^

Similar outcomes in this study were reported from a UK study that examined health visitors’ and nurses’ views in promoting physical activity in primary care settings.^31^ Ninety percent (*n* = 149) of health visitors and 88% (*n* = 186) of practice nurses said that they were very likely or likely to recommend all apparently healthy adult patients to take moderate exercise. Yet reasons for not giving physical activity advice included lack of time, lack of education and educational materials both for healthcare professionals and patients. Respondents to the survey in our study also reported similar reasons. Positively, research in Finland has shown that uptake of supervised physical activity is higher in older adults if healthcare advice is given by professionals, in particular physiotherapists, when compared to physicians or other healthcare professionals such as nurses.^32^

The results in our study indicate that physiotherapists have adequate knowledge to deliver advice on physical activity during an individual’s prosthetic rehabilitation. However, results have shown that prosthetists and other members of the prosthetic rehabilitation team also have awareness and knowledge of physical activity guidelines. It is proposed that prosthetists could, with the correct guidance, support and willingness, formally promote physical activity for health over the long-term course of prosthetic care.

### Strengths, limitations and future work

This survey captured a good response rate with representation across different groups of healthcare professionals and across the United Kingdom including England, Northern Ireland, Scotland and Wales. The findings, however, are specific to the United Kingdom and may not be generalisable to other countries. It should be acknowledged that not all the views of professionals working with people with limb absence were captured. It would therefore be reasonable to assume that those who did respond may have been interested and more motivated towards participating in and promoting physical activity for health. The survey was designed with prescriptive questions which led the participant through focussed survey sections. Therefore, some questions may have prompted responses in subsequent questions, rather than the participant answering with completely independent thought. Positively, the free-responses section allowed for liberal thought. Future research using a qualitative approach might yield more deeper and detailed responses.

## Conclusion

Promoting an active lifestyle should be an important component of care for people with lower limb absence, given that the majority of patients have underlying vascular problems, are older and therefore likely to have low levels of physical activity. This study highlights a clear need to implement both pre- and post-qualification education and training on physical activity guidelines and promotion to all members of the rehabilitation care team. Future research should explore the views of post-qualification prosthetists, and undergraduate prosthetic and orthotic students on the preferred mode of knowledge exchange on the topics of physical activity guidelines and promotion.

## Supplemental Material

10.1177_0309364620920109_Supplementary_File_1 – Supplemental material for Physical activity guidelines and promotion: An online survey of United Kingdom’s prosthetic rehabilitation healthcare professionals Click here for additional data file. Supplemental material, 10.1177_0309364620920109_Supplementary_File_1 for Physical activity guidelines and promotion: An online survey of United Kingdom’s prosthetic rehabilitation healthcare professionals by Sarah Deans, Alison Kirk, Anthony McGarry and David Rowe in Prosthetics and Orthotics International

10.1177_0309364620920109_Supplementary_File_2 – Supplemental material for Physical activity guidelines and promotion: An online survey of United Kingdom’s prosthetic rehabilitation healthcare professionals Click here for additional data file. Supplemental material, 10.1177_0309364620920109_Supplementary_File_2 for Physical activity guidelines and promotion: An online survey of United Kingdom’s prosthetic rehabilitation healthcare professionals by Sarah Deans, Alison Kirk, Anthony McGarry and David Rowe in Prosthetics and Orthotics International

10.1177_0309364620920109_Supplementary_File_3 – Supplemental material for Physical activity guidelines and promotion: An online survey of United Kingdom’s prosthetic rehabilitation healthcare professionals Click here for additional data file. Supplemental material, 10.1177_0309364620920109_Supplementary_File_3 for Physical activity guidelines and promotion: An online survey of United Kingdom’s prosthetic rehabilitation healthcare professionals by Sarah Deans, Alison Kirk, Anthony McGarry and David Rowe in Prosthetics and Orthotics International
