# Supplementary material for: Physical activity guidelines and promotion: An online survey of United Kingdom’s prosthetic rehabilitation healthcare professionals
Source: Prosthet Orthot Int. 2020 May 24;44(4):192–201. doi: 10.1177/0309364620920109 (PMC7594372; doi:10.1177/0309364620920109)
Supplement: 10.1177_0309364620920109_Supplementary_File_1 – Supplemental material for Physical activity guidelines and promotion: An online survey of United Kingdom’s prosthetic rehabilitation healthcare professionals [file 10.1177_0309364620920109_Supplementary_File_1.pdf]

Supplemental file 1 Definitive online survey version

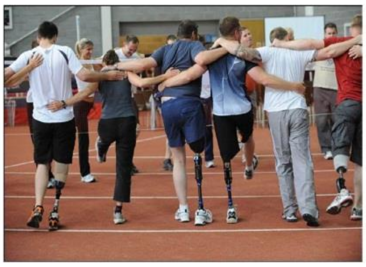

Survey Title
UK health professionals who care for people with limb absence:
their knowledge, beliefs and behaviours regarding physical activity promotion.

Thank you for your interest in this survey. Understanding your knowledge of physical activity guidelines and your views on physical activity promotion is extremely important to us.

The survey focuses on physical activity for maintaining or improving health in people who have limb absence.

Please keep in mind the idea of physical activity for maintaining or improving health rather than for sports participation or fitness benefits.

The survey should take ten minutes to complete and there is an opportunity for you to be entered into an optional free prize draw to win an iPod Shuffle on completion of the survey.

The survey covering letter and participant information sheet can be viewed by clicking on the links below.

Survey covering letter   Participant information sheet

If you have read the covering letter and information sheet, please tick whether you would like to take part in our survey.

- ○ Yes, I would like to take part
- ○ No, I've decided not to take part

Part I is about your current knowledge and understanding of physical activity guidelines for the general population.

Please answer the questions to the best of your knowledge.

Q1. ARE YOU AWARE THERE ARE PHYSICAL ACTIVITY GUIDELINES? (please select one)

- ○ Yes (if you know the name of the guideline and/or country of publication, please note it here) __________
- ○ No

Q2. ARE YOU AWARE OF THE CONTENT OF PHYSICAL ACTIVITY GUIDELINES? (please select one)

- ○ Yes
- ○ No

Q3. FROM WHICH SOURCE DID YOU LEARN ABOUT PHYSICAL ACTIVITY GUIDELINES? (please select all that apply)

- ☐ On-line learning
- ☐ Higher education course
- ☐ Work-based seminar
- ☐ Self-directed learning
- ☐ Published articles
- ☐ Other (please describe - optional) __________

Q4. DO YOU THINK BEING PHYSICALLY ACTIVE IS NECESSARY TO MAINTAIN OR IMPROVE OVERALL HEALTH? (please select one)

- Yes
- No

Q5. WHAT IS THE MINIMUM NUMBER OF DAYS PER WEEK A PERSON MUST BE PHYSICALLY ACTIVE IN ORDER TO IMPROVE OR MAINTAIN OVERALL HEALTH? (please select one)

- 0
- 1
- 2
- 3
- 4
- 5
- 6
- 7

Q6. WHAT IS THE MINIMUM INTENSITY OF PHYSICAL ACTIVITY NECESSARY TO MAINTAIN OR IMPROVE OVERALL HEALTH? (please select one)

- Very light
- Light
- Moderate
- Vigorous
- Very vigorous

Q7. IF A PERSON DOES ONLY MODERATE INTENSITY PHYSICAL ACTIVITY, FOR HOW MANY MINUTES SHOULD THIS TOTAL PER WEEK IN ORDER TO MAINTAIN OR IMPROVE HEALTH? (please select one)

- At least 60
- At least 75
- At least 90
- At least 105
- At least 120
- At least 135
- At least 150

Q8. WHICH ONE OF THE FOLLOWING CONSTITUTES MODERATE INTENSITY PHYSICAL ACTIVITY? (please select one)

- Casual walking
- Brisk walking
- Jogging
- Running
- Sprinting
- None of the above

Q9. WHICH ONE OF THE FOLLOWING CONSTITUTES VIGOROUS INTENSITY PHYSICAL ACTIVITY? (please select one)

- Casual walking
- Brisk walking
- Jogging
- None of the above

Q10. WHEN COMPARED TO MODERATE INTENSITY PHYSICAL ACTIVITY, DO YOU THINK COMPARABLE HEALTH BENEFITS CAN BE ACHIEVED THROUGH VIGOROUS INTENSITY ACTIVITY? (please select one)

- Yes
- No

Q11. FOR HOW MANY MINUTES OVER A WEEK DO YOU THINK VIGOROUS INTENSITY PHYSICAL ACTIVITY SHOULD BE PERFORMED IN ORDER TO ACHIEVE COMPARABLE HEALTH BENEFITS TO PERFORMING MODERATE INTENSITY PHYSICAL ACTIVITY? (please select one)

- At least 60
- At least 75
- At least 90
- At least 105
- At least 120
- At least 135
- At least 150

Q12. DO YOU THINK PEOPLE SHOULD UNDERTAKE PHYSICAL ACTIVITY TO MAINTAIN OR IMPROVE MUSCLE STRENGTH? (please select one)

- Yes
- No

Q13. ON HOW MANY DAYS A WEEK DO YOU THINK PEOPLE SHOULD PARTICIPATE IN MUSCLE STRENGTHENING ACTIVITIES? (please select one)

- 0
- 1
- 2
- 3
- 4
- 5
- 6
- 7

Q14. DO YOU THINK PEOPLE SHOULD UNDERTAKE PHYSICAL ACTIVITY TO MAINTAIN OR IMPROVE JOINT FLEXIBILITY? (please select one)

- Yes
- No

Q15. ON HOW MANY DAYS A WEEK DO YOU THINK PEOPLE SHOULD PARTICIPATE IN FLEXIBILITY ACTIVITIES? (please select one)

- 0
- 1
- 2
- 3
- 4
- 5
- 6
- 7

PART II is about your current practice related to physical activity promotion for people with limb absence (referred to here as patients).

Please tick one answer to indicate the extent to which you agree with the following statements.

Q16. I PROMOTE PHYSICAL ACTIVITY TO PATIENTS (please select one)

- Always
- Most of the Time
- Sometimes
- Rarely
- Never

Q17. I ENJOY PROMOTING PHYSICAL ACTIVITY TO PATIENTS (please select one)

- Always
- Most of the Time
- Sometimes
- Rarely
- Never

Q18. I HAVE TIME TO PROMOTE PHYSICAL ACTIVITY TO PATIENTS (please select one)

- Always
- Most of the Time
- Sometimes
- Rarely
- Never

Q19. I HAVE ADEQUATE KNOWLEDGE TO BE ABLE TO PROMOTE PHYSICAL ACTIVITY TO PATIENTS (please select one)

- Always
- Most of the Time
- Sometimes
- Rarely
- Never

Q20. I AM CONFIDENT ABOUT PROMOTING PHYSICAL ACTIVITY TO PATIENTS (please select one)

- Always
- Most of the Time
- Sometimes
- Rarely
- Never

Q21. OTHER HEALTH AND SOCIAL CARE PROFESSIONALS SHOULD PROMOTE PHYSICAL ACTIVITY TO PATIENTS (please select one)

- Always
- Most of the Time
- Sometimes
- Rarely
- Never

Q22. I DISCUSS PHYSICAL ACTIVITY PROMOTION WITH OTHER HEALTH & SOCIAL CARE PROFESSIONALS (please select one)

- Always
- Most of the Time
- Sometimes
- Rarely
- Never

Q23. MY WORKPLACE MANAGEMENT EXPECTS ME TO PROMOTE PHYSICAL ACTIVITY TO PATIENTS (please select one)

- Always
- Most of the Time
- Sometimes
- Rarely
- Never

Q24. MY PROFESSIONAL ASSOCIATION ENCOURAGES ME TO PROMOTE PHYSICAL ACTIVITY TO PATIENTS (please select one)

- Always
- Most of the Time
- Sometimes
- Rarely
- Never

Q25. I HAVE UNDERTAKEN PRE-QUALIFICATION LEARNING ON THE TOPIC OF PHYSICAL ACTIVITY PROMOTION (please select one)

- Always
- Most of the Time
- Sometimes
- Rarely
- Never

Q26. I HAVE UNDERTAKEN OR AM UNDERTAKING POST-QUALIFICATION LEARNING ON THE TOPIC OF PHYSICAL ACTIVITY PROMOTION (please select one)

- Always
- Most of the Time
- Sometimes
- Rarely
- Never

PART III is about your attitudes and beliefs towards what you consider to be desirable practice in promoting physical activity to people with limb absence (again referred to here as patients).

Please tick one answer to indicate the extent to which you agree with the following statements.

Q27. I SHOULD PROMOTE PHYSICAL ACTIVITY TO PATIENTS (please select one)

- Strongly agree
- Agree
- Neither Agree nor Disagree
- Disagree
- Strongly Disagree

Q28. MY WORKPLACE MANAGEMENT SHOULD EXPECT ME TO PROMOTE PHYSICAL ACTIVITY TO PATIENTS (please select one)

- Strongly agree
- Agree
- Neither Agree nor Disagree
- Disagree
- Strongly Disagree

Q29. MY PROFESSIONAL ASSOCIATION SHOULD ENCOURAGE ME TO PROMOTE PHYSICAL ACTIVITY TO PATIENTS (please select one)

- Strongly agree
- Agree
- Neither Agree nor Disagree
- Disagree
- Strongly Disagree

Q30. OTHER HEALTH AND SOCIAL CARE PROFESSIONALS SHOULD PROMOTE PHYSICAL ACTIVITY TO PATIENTS (please select one)

- Strongly agree
- Agree
- Neither Agree nor Disagree
- Disagree
- Strongly Disagree

Q31. PRE-QUALIFICATION HEALTH AND SOCIAL CARE STUDENTS SHOULD BE EDUCATED AT HIGHER EDUCATION LEVEL ON PATIENT PHYSICAL ACTIVITY PROMOTION (please select one)

- Strongly agree
- Agree
- Neither Agree nor Disagree
- Disagree
- Strongly Disagree

Q32. CONTINUING PROFESSIONAL DEVELOPMENT (CPD) COURSES SHOULD EXIST ON PATIENT PHYSICAL ACTIVITY PROMOTION (please select one)

- Strongly agree
- Agree
- Neither Agree nor Disagree
- Disagree
- Strongly Disagree

Q33. I WOULD ATTEND PATIENT PHYSICAL ACTIVITY PROMOTION CPD COURSES IF THEY WERE AVAILABLE (please select one)

- Strongly agree
- Agree
- Neither Agree nor Disagree
- Disagree
- Strongly Disagree

And finally, PART IV of the survey has six quick questions about you.

Q34. WHAT IS YOUR PROFESSIONAL TITLE? (please select all that apply)

- Prosthetist/Orthotist
- Prosthetist
- Orthotist
- Consultant in Rehabilitation Medicine
- Physiotherapist
- Nurse
- Another health and social care professional (please describe – optional)

Q35. WHAT IS YOUR GENDER? (please select one)

- Male
- Female

Q36. WHAT IS YOUR AGE? (please select one)

- 20–30
- 31–40
- 41–50
- 51–60
- Over 60

Q37. FOR HOW MANY YEARS HAVE YOU BEEN QUALIFIED? (please select one)

- 0–10
- 11–20
- 21–30
- 31–40
- Over 40

Q38. FOR HOW MANY YEARS HAVE YOU BEEN WORKING IN CLINICAL PRACTICE? (please select one)

- 0–10
- 11–20
- 21–30
- 31–40
- Over 40

Q39. WHAT IS THE GEOGRAPHICAL LOCATION OF YOUR USUAL PLACE OF WORK? (please select one)

- England
- Northern Ireland
- Scotland
- Wales

Q40. We appreciate you may have other thoughts, ideas, opinions and views which we haven't covered elsewhere in the survey.

We would be very interested in these and would really like you to note them down in the following space.

Thank you so much for taking the time to complete this survey. We are grateful for your responses which will always be treated confidentially.

By way of a thank–you for completing the survey you are invited to enter into our free draw to win an iPod Shuffle. Please enter your email address here if you wish to be entered into the draw.

We're sorry that you have chosen not to take part in the survey but thanks for considering it anyway.
