# Supplementary material for: Physical activity guidelines and promotion: An online survey of United Kingdom’s prosthetic rehabilitation healthcare professionals
Source: Prosthet Orthot Int. 2020 May 24;44(4):192–201. doi: 10.1177/0309364620920109 (PMC7594372; doi:10.1177/0309364620920109)
Supplement: 10.1177_0309364620920109_Supplementary_File_2 – Supplemental material for Physical activity guidelines and promotion: An online survey of United Kingdom’s prosthetic rehabilitation healthcare professionals [file 10.1177_0309364620920109_Supplementary_File_2.pdf]

Supplemental file 2 - Correct answers to items examining respondents' knowledge and understanding of the UK physical activity guidelines

Item	Correct answer
Q5 What is the minimum number of days per week a person must be physically active in order to improve or maintain overall health?	5 days/week
Q6 What is the minimum intensity of physical activity necessary to maintain or improve overall health?	Moderate
Q7 If a person does only moderate intensity physical activity, for how many minutes should this total per week in order to maintain or improve health?	150 minutes
Q8 Which one of the following constitutes moderate intensity physical activity?	Brisk walking (or jogging)
Q9 Which one of the following constitutes vigorous intensity physical activity?	Jogging (or brisk walking)
Q10 When compared to moderate intensity physical activity, do you think comparable health benefits can be achieved through vigorous intensity activity?	Yes
Q11 For how many minutes over a week do you think vigorous intensity physical activity should be performed in order to achieve comparable health benefits to performing moderate intensity physical activity?	At least 75 minutes
Q12 Do you think people should undertake physical activity to maintain or improve muscle strength?	Yes
Q13 On how many days a week do you think people should participate in muscle strengthening activities?	2 days/week
Q14 Do you think people should undertake physical activity to maintain or improve joint flexibility?	Yes
Q15 On how many days a week do you think people should participate in flexibility activities?	2 days/week
