# Supplementary material for: Physical activity guidelines and promotion: An online survey of United Kingdom’s prosthetic rehabilitation healthcare professionals
Source: Prosthet Orthot Int. 2020 May 24;44(4):192–201. doi: 10.1177/0309364620920109 (PMC7594372; doi:10.1177/0309364620920109)
Supplement: 10.1177_0309364620920109_Supplementary_File_3 – Supplemental material for Physical activity guidelines and promotion: An online survey of United Kingdom’s prosthetic rehabilitation healthcare professionals [file 10.1177_0309364620920109_Supplementary_File_3.pdf]

Supplemental file 3 - Categorised text entry responses to open survey question

Category/theme	Statement
Respondents' acknowledgement of the levels of physical activity within their own health professional community<br><br>and<br><br>Healthcare professionals as role models	• I think much depends on the clinician's individual participation in exercise - it would be interesting to ask how much and what sort of exercise they complete each week (physiotherapist)<br>• It might be interesting to see how much physical activity practitioners themselves partake in. Do we practise what we preach? (physiotherapist)<br>• I try and promote physical activity with my patients with limb loss but accessibility and motivation are often a problem (physiotherapist)<br>• Most health care workers don't follow 'the guidelines'! (prosthetist/orthotist)<br>• I do enforce with patients the importance of activity and exercise but realise that for many, many patients it is a difficult challenge even getting in and out of bed unassisted (prosthetist/orthotist)<br>• I think my own inactivity has held me back from fully engaging in promoting physical activity to prosthetic limb users. I feel I could have been more confident in this if I was more active myself (prosthetist/orthotist)
Comorbidity in people with limb absence	• One barrier to my confidence in promoting physical activity is concern about exercise adversely affecting any condition the patient may have (prosthetist)<br>• Promoting physical activity with not only limb loss but multi-pathologies including heart conditions can be difficult, if they have been advised by other health professional to take things easy. Also family members often still believe that physical activity may detrimental (physiotherapist)<br>• Many elderly patients lack the mental capacity too to take on board the importance of keeping on the move. Many people STOP activity when their heart rate is increased, because they FEAR it - some will have had angina or heart problems previously (prosthetist/orthotist)<br>• Many of our patients have other comorbidities that it is very difficult to lecture them on exercise when they have such reduced mobility (prosthetist)<br>• Clearly for most individuals exercise is essential to maintain or increase health but we do have patients whose vascular systems are severely compromised and therefore should have medical advice prior to commencing exercise. For these individuals very careful advice is required (prosthetist)<br>• In some cases where there are other complications e.g. cardio-vascular implications or safety concerns/other pathologies, it may even be necessary to reduce a patient's activity expectations. Understanding this is just as important if not more, than promotion of exercise (prosthetist/orthotist)<br>• Obviously amputees, like other sections of the community, can have special needs/considerations to take into account, but please don't isolate amputees (or orthotic users etc.) from the wider population

	(prosthetist/orthotist)<br>• Pushing a wheelchair is a hard enough challenge for the vast majority of patients that we may see (prosthetist/orthotist)
Prosthetic prescription	• Imperfect prosthetic provision will also contribute to patients' ability to be more mobile (prosthetist/orthotist)<br>• I feel there is a lack of activity options for lower limb amputees who are not limb fitted (physiotherapist)<br>• The NHS does not support the provision of specific leisure of sport activity limb. Arguably many activities may be pursued without the need for a specifically designed prosthesis (prosthetist/orthotist)<br>• The other drawback is prosthetic prescription for the more active patients. With budgetary restrictions, it is more difficult to provide a prosthesis for recreational use (such as swimming) which would help increase/improve patients' fitness (prosthetist/orthotist)
Respondents' views on the role/s of other healthcare professionals	• I feel that the GP has a better overall view of the patient's health and therefore is in a better position to promote physical activity within the patient's limitations (prosthetist)<br>• Healthcare and social care professionals should only give advice re physical activity if they have the relevant experience and background knowledge. Not ALL exercises are suitable for ALL of the population and incorrect advice/exercise can be unbeneficial or in extreme cases cause injury/damage (physiotherapist)<br>• Promoting physical activity with not only limb loss but multi-pathologies including heart conditions can be difficult, if they have been advised by other health professional to take things easy. Also family members often still believe that physical activity may detrimental (physiotherapist)<br>• Not enough emphasis placed on prevention and personal responsibility by large proportion of general population and/or medical profession (physiotherapist)<br>• Promotion of physical activity is/should be one of the central pillars of physiotherapy treatment (physiotherapist)<br>• The NHS however has no HCP [healthcare professional] group charged with delivering this message and as such is reliant on individuals. Some professional groups pretend to be the experts but rarely have the level of training to deliver in this area (medic)<br>• I am also an amputee so I talk to my patients about the type of exercise that works for me, hoping it will encourage my patients (prosthetist/orthotist)<br>• There is a GP [General Practitioner] exercise referral scheme available which some patients do access (prosthetist/orthotist)<br>• Clinical professionals could effectively work with sports coaches etc to promote physical and emotional health (coach in rehabilitation)
